# *Aspergillus* in Italian Pistachios: Characterization and Detection of Major Aflatoxigenic Species With a Loop‐Mediated Isothermal Amplification Assay

**DOI:** 10.1002/fsn3.71146

**Published:** 2025-10-29

**Authors:** Wanissa Mellikeche, Alessandra Ricelli, Marah Abukhmaish, Rocco Caracciolo, Marilita Gallo, Carlotta Casini, Giancarlo Colelli, Anna Maria D'onghia, Franco Valentini

**Affiliations:** ^1^ Department of Agricultural Sciences, Food, Natural Resources and Engineering University of Foggia Foggia Italy; ^2^ International Centre for Advanced Mediterranean Agronomic Studies Valenzano (BA) Italy; ^3^ National Research Council Institute of Molecular Biology and Pathology Rome Italy; ^4^ Enbiotech SRL Angri (SA) Italy; ^5^ Department of Diagnostics and Public Health University of Verona Verona Italy

**Keywords:** *A. flavus*, aflatoxins, LAMP, mycotoxins, nuts, postharvest

## Abstract

Aflatoxigenic *Aspergillus* species are major postharvest contaminants of pistachio nuts, producing harmful aflatoxins that are strictly regulated by most countries. Therefore, their accurate detection is crucial for effective control strategies. Loop‐mediated isothermal amplification (LAMP) is a simple molecular technique that uses 4–6 primers to achieve high sensitivity and specificity through a thermostatic amplification reaction. This study aimed to isolate *Aspergillus* spp. (section *Flavi*) from pistachios sampled in Italy and to determine their capacity to produce Aflatoxin B1 (AFB1). It revealed a high occurrence of *A. flavus* , followed by *A. parasiticus* . Furthermore, all isolates identified as these species exhibited AFB1 production, reaching 10107.8 and 1448.2 ng/mL for *A. flavus* PGA95 and *A. parasiticus* PGA72, respectively. Based on these results, a LAMP assay was developed to detect these species by targeting the *nor1* gene, involved in the aflatoxin production pathway. The assay demonstrated a sensitivity of 0.03 pg of DNA per reaction and high specificity in detecting only targeted species. To simplify the assay and enhance its applicability, primers were air‐dried at 30°C to preserve stability and eliminate the need for repeated reagent preparation. Dehydrated primers maintained their stability for up to 45 days of storage even at 35°C. Additionally, the LAMP assay successfully detected fungi directly on pistachios at contamination levels as low as 0.5 spores/g. This study identifies prevalent aflatoxigenic *Aspergillus* species in Italian pistachios and introduces LAMP as a rapid, sensitive, and specific detection tool.

## Graphical abstract

Identification *A. flavus* and *A. parasiticus* was done as major aflatoxigenic contaminants in Italian pistachios, both capable of producing high levels of Aflatoxin B1. Development of a LAMP assay targeting the nor1 gene was done, showing high sensitivity (0.03 pg of DNA) and specificity, even detecting contamination as low as 0.5 spores/g. Primers enhanced assay stability and usability, remaining effective for up to 45 days at 35°C.

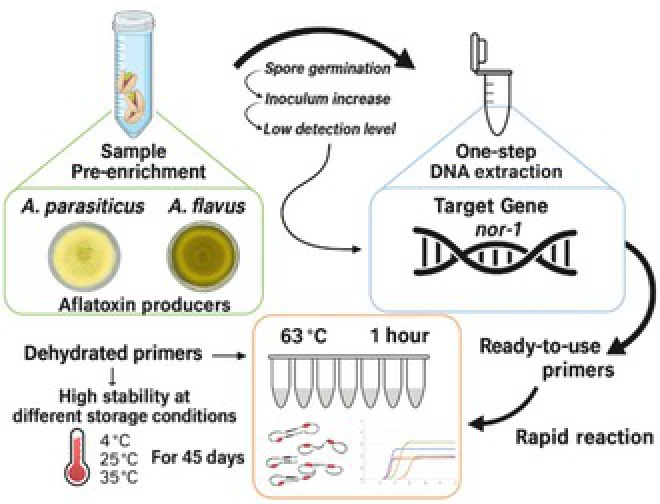

## 1 Introduction

Before pistachio nuts arrive at market shelves, bringing their distinct flavors and high nutritional value to our daily diets, they must undergo testing and analysis to ensure that their aflatoxin content is below tolerable limits. Aflatoxins are naturally occurring mycotoxins, which are produced mainly by *Aspergillus* molds and pose serious health risks to consumers. There are four main types of aflatoxins (AFB1, AFB2, AFG1, and AFG2) of which AFB1 is the most dangerous one. Consequently, most regulations target not only total aflatoxin content but AFB1 specifically. In Europe, pistachios intended for direct consumption cannot be imported and marketed if they contain more than 8 μg/g of AFB1 and/or 10 μg/g total aflatoxins. Furthermore, treatments available for mycotoxins are very limited. Therefore, farmers in producing countries must take all the precautions necessary to avoid a highly contaminated final product mainly by targeting aflatoxigenic fungal species. These belong to the *Aspergillus* sections *Flavi*, *Nidulantes*, and *Ochraceorosei* (Varga et al. 2009; Varga et al. 2011). The *Flavi* section is the one most implicated in the production of aflatoxins because the majority of section's 33 species are producers of aflatoxins and, among these, *A. parasiticus* and *A. flavus* are the most economically relevant.

Aflatoxigenic *Aspergillus* spp. are detected on a variety of nuts but particularly on pistachios. These contaminations can occur when nuts are still on the tree or after harvesting and during storage and distribution. The presence of *Aspergillus* molds can significantly increase after harvesting and drying due to their tolerance to low humidity levels.

The reduction of *Aspergillus* inoculum in the field and postharvest can significantly affect the final contamination level. However, early and rapid detection and continuous monitoring of the pathogens are necessary, using mass testing methods that are fast, easy to apply, affordable for mass testing, and sensitive enough to provide accurate results. One method that meets these requirements is loop‐mediated isothermal amplification (LAMP), developed by Notomi et al. (2000). It uses four to six primers to target a DNA region and amplify it at a fixed temperature. The LAMP detection allows the screening of pistachio samples during and after postharvest processing in order to evaluate the inoculum and decide whether treatment is necessary.

LAMP has been previously developed to detect several postharvest molds including *Aspergillus* spp. (Mellikeche et al. 2022). The studies of Luo et al. (2012) designed specific LAMP assays for the detection of aflatoxigenic aspergilli associated with Brazil nuts including *A. nomius* and *A. parasiticus* or *A. flavus* . These assays were further tested on several foods in the study of Luo et al. (2014), but not on pistachios despite their susceptibility to the targeted contaminants. Niessen et al. (2018) also developed a LAMP assay targeting the *nor1* gene in aflatoxin‐producing *Aspergillus* section *Flavi*, although their approach was not optimized for pistachios, and relied on primers in liquid form without stabilization, using more methods for result detection. This study aims to isolate and characterize aflatoxigenic aspergilli associated with Italian pistachios and to develop a unique real‐time LAMP assay to detect these species. This assay targets the *nor1* gene, which is directly linked to aflatoxin production pathways (Yu et al. 2004) in species belonging to the *Aspergillus* section *Flavi*. To further simplify the assay and facilitate its use in field and low‐resource settings, the study also incorporates the preparation of ready‐to‐use, air‐dried primer mixes with higher stability and longer shelf life.

## 2 Material and Methods

### 2.1 Fungal Strains

Fungal strains of *A. flavus* , *A. tamarii*, *A. niger* , *A. tubingensis*, and *Penicillium digitatum* were used to study the effectiveness and specificity of the primers. All these strains were isolated from nuts and belong to the CIHEAM Bari fungal collection (Mellikeche, Ricelli, et al. 2024).

### 2.2 Sampling and Isolation of Toxigenic *Aspergillus* spp. Sec. *Flavi*

Six samples of 1 kg each of dried pistachios (cultivar Bianca) were obtained from a pistachio storage warehouse in Bronte, Italy. An isolation step was performed before LAMP primer design to determine the population of *Aspergillus* sec. *Flavi* associated with Italian pistachios and the species to target with the LAMP assay.

The isolation of *Aspergillus* spp. was performed following the method described by Mellikeche, Casini, et al. (2024). Briefly, a spore suspension was prepared by adding 50 mL of 0.1% Tween20 to 50 pistachio nuts and shaking for 1 h in an orbital shaker (120 rpm). The resulting suspension was used directly and after a 10‐fold dilution, 100 μL was spread on Petri dishes containing freshly prepared medium and incubated at 25°C. The medium chosen for this isolation was *Aspergillus* Differentiation Agar (Merck, UK), which allowed the growth of *Aspergillus* spp. sec. *Flavi* as distinctly pink colonies. These were then transferred into potato dextrose agar (PDA) and left to grow at 25°C. The isolates were then purified into single‐spore colonies that were transferred to potato dextrose broth (PDB), a liquid medium from which DNA was extracted.

### 2.3 DNA Extraction

Pure DNA of each fungal strain was extracted by the method of Carlucci et al. (2013) from four‐day‐old mycelium grown in PDB at 25°C in an orbital shaker.

Crude DNA extraction from contaminated nut samples was carried out directly from 4 g of inoculated pistachio nuts following a 24‐h pre‐enrichment period in 36 mL of PDB at 25°C. The liquid medium was filtered through Whatman n°4 paper (47 mm diameter) using a filtration ramp to capture the germinated spores. The filter was then incubated in 500 μL of a one‐step extraction buffer (Enbiotech S.r.l) at 95°C for 10 min.

### 2.4 Identification of Isolated *Aspergillus* spp.

All isolates were tested with *A. flavus* ‐specific LAMP primers designed and validated by Mellikeche, Ricelli, et al. (2024). Isolates that tested negative were further identified using PCR amplification of the calmodulin (CaM) and internal transcribed spacer (ITS) regions. The resulting PCR products were sequenced, and the sequences were compared against the NCBI GenBank database using BLASTn for species‐level identification. A multi‐locus sequence identification approach was employed, combining both CaM and ITS sequences to improve accuracy.

### 2.5 AFB1 Production

The capacities of the isolated aspergilli to produce aflatoxin were confirmed by high‐performance thin‐layer chromatography (HPTLC) and high‐performance liquid chromatography (HPLC) analysis following the protocol described by Mellikeche, Mellikeche, Casini, et al. (2024). Three repetitions of both analyses were done for each isolate.

### 2.6 Primer Design

**FIGURE 1:**
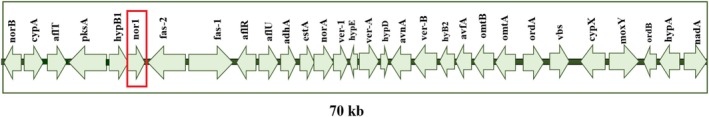
Aflatoxin gene cluster for *A. flavus* (Mellikeche, Ricelli, et al. 2024).

The aim of this step was to design generic primers that target a specific region of the gene *nor1* (norsolorinic acid gene) that belongs to the aflatoxin cluster (Figure 1) and is directly involved in their biosynthesis (Yu et al. 2004). The chosen region must be shared by all targeted aflatoxigenic aspergilli. Therefore, gene sequences of targeted species obtained from the NCBI gene database (https://www.ncbi.nlm.nih.gov/gene/) were aligned accordingly using the Clustal Omega tool (https://www.ebi.ac.uk/Tools/msa/clustalo/). LAMP primer sets containing two outer primers (F3 and B3) and two inner primers (FIP and BIP) were designed using Primer Explorer 5, with default settings provided by Eiken Chemical Co. Ltd. Tokyo, Japan (http://primerexplorer.jp/lampv5e/index.html). In order to increase the rapidity and sensitivity of the LAMP reaction, additional loop primers (LF and LB) were manually designed for each set.

Minor modifications were made to some primer sequences to reduce dimer formation and optimize melting temperatures. Primer efficiency was subsequently verified through sensitivity and specificity testing.

In silico analysis of LAMP primers targeting the *nor1* gene was performed using BLASTn against NCBI nucleotide databases. Primer sequences were assessed for specificity to *Aspergillus flavus* and *Aspergillus parasiticus*. Sequence alignments confirmed target binding sites and evaluated potential cross‐reactivity with nontarget species.

### 2.7 LAMP Reaction and Result Visualization

For each reaction, the DNA was added to the designed primers, a LAMP mix (Enbiotech Srl, Italy) containing a strand‐displacing DNA polymerase, dNTPs, and salts needed for the amplification and double‐strand intercalating DNA‐binding dye EvaGreen (Biotium Inc., USA). The latter binds to the double‐stranded DNA and emits a fluorescence signal that increases throughout the amplification. This detection is done in real time using a specific LAMP device provided by ICgene (Enbiotech Srl, Italy) and linked to a smart tablet with the ICgene application installed that allows real‐time interpretation and visualization of results.

### 2.8 Optimization of the LAMP Assay Conditions

LAMP primer sets were initially tested on the DNA of AFB1‐producing *A. flavus* strain BP53 and *A. parasiticus* strain AF2, and the best‐performing set was selected for optimization. This process refers to the adjustment of the various parameters involved in the reaction to achieve the best possible performance of the assay. These parameters include the reagent concentrations and the determination of their optimal temperature after repetition at 55°C, 60°C, and 63°C.

The primers' specificity was tested by targeting available strains that are phylogenetically close to the targets as well as *Aspergillus* strains, which are known for their inability to produce aflatoxins. Sensitivity was determined by testing 10‐fold dilutions of pure DNA of target isolates.

### 2.9 Primer Dehydration and Stability

Primer dehydration is an efficient technique for enhancing assay performance, facilitating the primers' use and maintaining their stability during storage. After determination of the suitable concentrations and optimal reaction conditions, mixes of the primers and the dye were prepared in 0.2‐mL disposable tubes and dehydrated. This was performed by using a centrifugal concentrator (Labconco, USA). For a dehydration cycle of 15 min, two temperatures were tested: 60°C and 30°C. The optimal temperature for dehydration was further used in subsequent tests.

Initial stability tests were conducted immediately after dehydration and compared with freshly prepared primer solutions. The tests were repeated after 15 days of storage at room temperature (RT) to ensure that the technique remained effective over time. A total of six samples were tested: one positive control and five negative controls. This setup was designed to confirm that the mixing and dehydration processes did not cause the LAMP assay to produce false‐positive results.

After choosing the best dehydration temperature, primers' stability was evaluated over a 45‐day period under different storage temperatures: 4°C, RT (25°C ± 2°C), and 35°C, with assessments carried out every 15 days. This was done to assess primer stability under varying storage conditions, including scenarios where refrigeration or other controlled storage equipment may not be available, such as during transportation or use in resource‐limited settings. These tests included 2 positive controls (DNA of *A. flavus* and *A. parasiticus* at a concentration of 0.01 ng/mL) and 8 negative controls.

### 2.10 Evaluation of the Primer Sets on Food Material

Pistachios were chosen as the matrix for LAMP assay implementation. In order to control the inoculum and accurately determine the assay's level of detection, pistachios used for this test were first sterilized and then artificially inoculated. Sterilization was performed by dipping in hypochlorite solution (1% chlorine) for 3 min, rinsing with plenty of sterile distilled water, and drying at 50°C overnight. Samples of 4 g of pistachios were then inoculated with 1 mL of *A. flavus* BP53 and *A. parasiticus* PGA72 spores' suspensions of 2, 10, and 100 spore/mL, and then left to stabilize at 4°C for 24 h. The inoculation was followed by a pre‐enrichment step, which allowed spore germination and mycelium production, thereby providing a more important quantity of DNA extracted (as previously explained). Pre‐enrichment was done by adding 36 mL of PDB and incubating at 25°C for 24 h. Two controls were considered for each experiment: non‐inoculated sterilized pistachios and, in the absence of pistachios, PDB with the inoculum.

To demonstrate the applicability of the assay, the optimally air‐dried primer set was tested using the established protocol on several pistachio varieties obtained from various sources, including the field, storage facilities, and local markets, as detailed in Table 1. For each sample, 4 g of pistachios was first surface‐sterilized and then artificially inoculated with a spore suspension of *A. flavus* at a concentration of 0.5 spores/g. The same setting was repeated for *A. parasiticus* as well. A positive control containing purified DNA and a negative control using nuclease‐free water were included for each test.

**TABLE 1:** Pistachio samples used to test the final LAMP assay.

Sample	Variety	Source
1	Kerman	Italian market (imported from the USA)
2	Kerman	Storage facility, Basilicata, Italy
3	Kerman	Field, Basilicata, Italy
4	Bianca (Napolitana)	Storage facility, Basilicata, Italy
5	Bianca (Napolitana)	Storage facility, Bronte, Italy
6	Aegina	Field, Basilicata, Italy
7	Aegina	Storage facility, Basilicata, Italy
8	Basilicata	Storage facility, Basilicata, Italy

## 3 Results

### 3.1 Isolation and Characterization of *Aspergillus* spp.

A total of 117 *Aspergillus* isolates showing green colonies on PDA were obtained from the pistachio samples. LAMP screening with specific *A. flavus* primers described by Mellikeche, Ricelli, et al. (2024) identified 109 of these as *Aspergillus flavus*, making it the most prevalent species at 93.13%. The remaining isolates consisted of five *A. parasiticus* and three *A. tamarii* (Table 2).

**TABLE 2:** Isolated strains and their AFB1 production.

Isolate	Species	AFB1/HPTLC	Average AFB1 production/HPLC (ng/ml)	S. D.
PGA1	*A. tamarii*	No	0.0	0.0
PGA2	*A. flavus*	Yes	74.0	4.4
PGA3	*A. flavus*	Yes	79.2	5.8
PGA4	*A. flavus*	Yes	123.0	7.4
PGA5	*A. flavus*	Yes	162,4	4.2
PGA6	*A. flavus*	Yes	84.7	3.9
PGA7	*A. flavus*	Yes	73.5	1.7
PGA8	*A. flavus*	Yes	263.4	13.1
PGA9	*A. flavus*	Yes	393.9	75.5
PGA10	*A. flavus*	Yes	120.4	9.0
PGA11	*A. flavus*	Yes	303.0	9.2
PGA12	*A. flavus*	Yes	210.7	7.3
PGA13	*A. flavus*	Yes	335.4	5.8
PGA14	*A. flavus*	Yes	294.5	8.3
PGA15	*A. flavus*	Yes	322.5	10.3
PGA16	*A. flavus*	Yes	321.9	10.5
PGA17	*A. flavus*	Yes	86.8	3.3
PGA18	*A. flavus*	Yes	361.1	18.3
PGA19	*A. flavus*	Yes	168.9	7.6
PGA20	*A. flavus*	Yes	477.6	16.4
PGA21	*A. flavus*	Yes	253.8	10.2
PGA22	*A. flavus*	Yes	309.3	15.7
PGA23	*A. flavus*	Yes	380.7	12.3
PGA24	*A. flavus*	Yes	257.6	13.3
PGA25	*A. flavus*	Yes	109.7	8.3
PGA26	*A. flavus*	Yes	131.9	7.3
PGA27	*A. flavus*	Yes	560.1	41.0
PGA28	*A. flavus*	Yes	530.4	64.8
PGA29	*A. flavus*	Yes	215.5	10.3
PGA30	*A. flavus*	Yes	225.7	9.7
PGA31	*A. tamarii*	No	0.0	0.0
PGA32	*A. flavus*	Yes	281.0	10.2
PGA33	*A. flavus*	Yes	232.4	8.7
PGA34	*A. flavus*	Yes	349.6	18.8
PGA35	*A. flavus*	Yes	225.6	10.5
PGA36	*A. flavus*	Yes	148.2	6.3
PGA37	*A. flavus*	Yes	116.2	5.8
PGA38	*A. flavus*	Yes	116.5	7.9
PGA39	*A. flavus*	Yes	283.9	9.9
PGA40	*A. flavus*	Yes	119.8	5.6
PGA41	*A. flavus*	Yes	252.5	14.2
PGA42	*A. flavus*	Yes	1404.7	18.3
PGA43	*A. flavus*	Yes	593.9	15.4
PGA44	*A. flavus*	Yes	444.2	16.8
PGA45	*A. flavus*	Yes	5719.8	300.9
PGA46	*A. flavus*	Yes	1154.4	43.9
PGA47	*A. flavus*	Yes	269.6	7.4
PGA48	*A. flavus*	No	0.0	0.0
PGA49	*A. flavus*	Yes	379.4	20.8
PGA50	*A. flavus*	Yes	184.4	7.0
PGA51	*A. flavus*	Yes	1535.3	53.1
PGA52	*A. flavus*	Yes	1898.3	85.5
PGA53	*A. flavus*	Yes	459.8	18.8
PGA54	*A. parasiticus*	Yes	571.9	23.6
PGA55	*A. flavus*	Yes	1007.0	59.8
PGA56	*A. parasiticus*	Yes	989.9	41.9
PGA57	*A. flavus*	Yes	330.7	9.3
PGA58	*A. flavus*	Yes	543.9	25.6
PGA59	*A. flavus*	Yes	404.7	14.9
PGA60	*A. flavus*	Yes	1245.2	57.2
PGA61	*A. flavus*	Yes	1162.9	44.5
PGA62	*A. flavus*	Yes	1037.7	40.7
PGA63	*A. flavus*	Yes	1229.4	52.4
PGA64	*A. flavus*	Yes	1463.7	38.8
PGA65	*A. flavus*	Yes	478.9	19.3
PGA66	*A. flavus*	Yes	839.1	32.1
PGA67	*A. flavus*	Yes	591.8	20.1
PGA68	*A. flavus*	Yes	422.0	13.8
PGA69	*A. flavus*	Yes	1366.9	46.3
PGA70	*A. parasiticus*	Yes	429.4	11.3
PGA71	*A. flavus*	Yes	4306.9	178.5
PGA72	*A. parasiticus*	Yes	1505.4	57.2
PGA73	*A. flavus*	Yes	709.7	14.9
PGA74	*A. flavus*	Yes	477.0	21.2
PGA75	*A. flavus*	Yes	341.6	16.6
PGA76	*A. flavus*	Yes	296.1	9.3
PGA77	*A. flavus*	Yes	814.1	8.3
PGA78	*A. flavus*	Yes	1635.3	144.1
PGA79	*A. flavus*	Yes	879.3	18.3
PGA80	*A. flavus*	Yes	391.6	12.4
PGA81	*A. flavus*	Yes	1917.8	88.0
PGA82	*A. flavus*	Yes	369.3	13.1
PGA83	*A. flavus*	Yes	3310.0	318,6
PGA84	*A. flavus*	Yes	1392.0	64,1
PGA85	*A. flavus*	Yes	6505.7	216,4
PGA86	*A. flavus*	Yes	2036.3	64.1
PGA87	*A. flavus*	Yes	699.6	28.6
PGA88	*A. tamarii*	No	0.0	0.0
PGA89	*A. flavus*	Yes	1061.3	41.1
PGA90	*A. flavus*	Yes	922.1	60.4
PGA91	*A. flavus*	Yes	585.7	26.8
PGA92	*A. parasiticus*	Yes	900.5	38.1
PGA93	*A. flavus*	Yes	2299.3	57.3
PGA94	*A. flavus*	Yes	156.7	5.8
PGA95	*A. flavus*	Yes	9926.0	181.8
PGA96	*A. flavus*	Yes	151.6	4.2
PGA97	*A. flavus*	Yes	390.8	20.6
PGA98	*A. flavus*	Yes	781.1	22.3
PGA99	*A. flavus*	Yes	1152.0	46.4
PGA100	*A. flavus*	Yes	1286.6	86.2
PGA101	*A. flavus*	Yes	1487.9	60.4
PGA102	*A. flavus*	Yes	479.8	18.4
PGA103	*A. flavus*	Yes	1054.4	54.6
PGA104	*A. flavus*	Yes	3687.7	205.4
PGA105	*A. flavus*	Yes	488.2	12.2
PGA106	*A. flavus*	Yes	521.8	16.4
PGA107	*A. flavus*	Yes	1158.8	53.2
PGA108	*A. flavus*	Yes	639.0	13.0
PGA109	*A. flavus*	Yes	842.2	28.6
PGA110	*A. flavus*	Yes	853.0	32.6
PGA111	*A. flavus*	Yes	890.9	29.1
PGA112	*A. flavus*	Yes	1021.0	30.2
PGA113	*A. flavus*	Yes	853.8	30.3
PGA114	*A. flavus*	Yes	41.9	1.3
PGA115	*A. flavus*	Yes	1304.25	23.9
PGA116	*A. flavus*	Yes	869.1	44.5
PGA117	*A. flavus*	Yes	338.45	19.8

Mycotoxin analysis showed that all *A. flavus* isolates were capable of producing AFB1, with certain strains producing exceptionally high levels. The overall production ranged from 41.9 to 9926.0 ng/mL, with isolates PGA114 and PGA95 showing the lowest and highest productions, respectively. Although *A. parasiticus* was significantly less frequent than *A. flavus* , its isolates demonstrated notable mycotoxin production, ranging from 424.4 to 1505.4 ng/mL by the isolates PGA70 and PGA72, respectively. In contrast, no AFB1 production was detected in any *A. tamarii* isolates.

### 3.2 LAMP Primer Design

**FIGURE 2:**
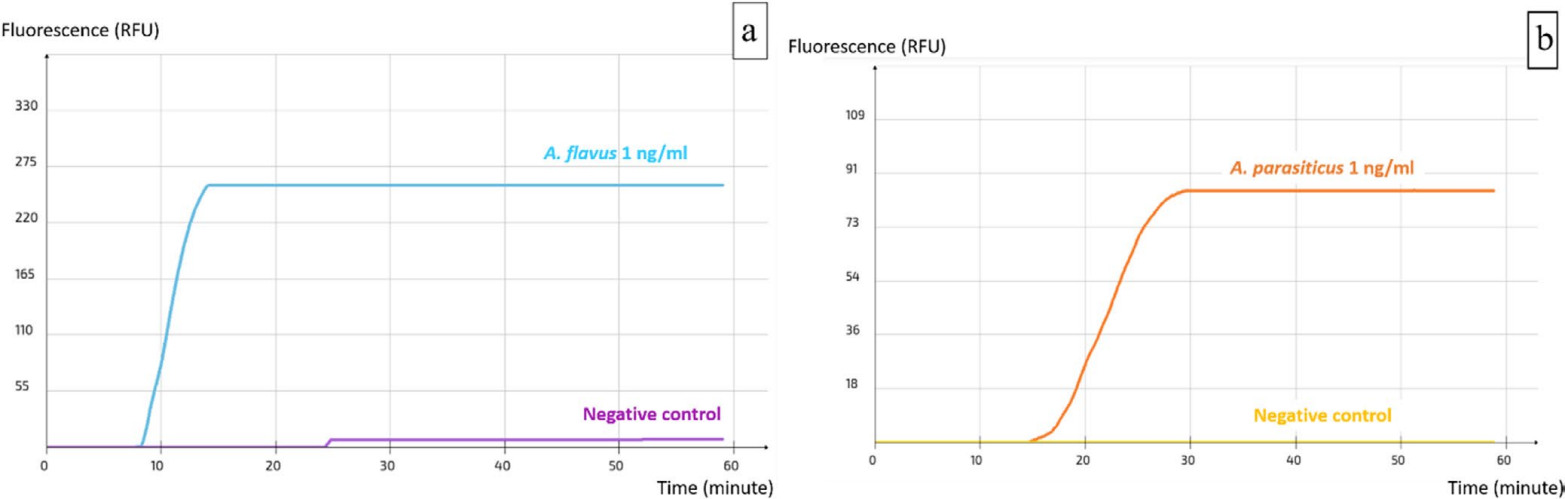
Visualization of the real‐time LAMP assay results with the primer set AFLS4 (a) *A. flavus* (b) *A. parasiticus* .

Primer design was done on several species; the presence of the target gene (*nor1*) in all considered species confirms their potential to produce AFB1. Primer Explorer V5 generated several suitable sets for the detection of targeted species. However, the alignment step was essential for modifying the primers to ensure that they specifically targeted *A. parasiticus* and *A. flavus* . During this step, loop primers were manually designed for the reaction's rapidity and specificity. Among the resulting sets, AFLS4 (Table 3) yielded the most promising and accurate results, effectively detecting specifically the targeted aflatoxin‐producing *Aspergillus* species. To visualize the assay results, the device set displayed a graph (Figure 2) in which a positive reaction is represented by a peak in the fluorescence signal, whose magnitude is independent of DNA quantity.

**TABLE 3:** Primer set AFLS4 for the detection of *A. flavus* and *A. parasiticus* .

Target	Primer name	Sequence	Tm (°C)	bp
*Aflatoxin‐producing Aspergillus* spp. *sec. Flavi* *Nor1*	AflS4‐F3	TGATGGTGCTGCTCGGG	57.6	17
	AflS4‐B3	GTGGTGGTTGCCAATGCG	58.2	18
	AflS4‐FIP	GCTCCCGTCCTACTGTTTCA‐TTTT‐CAAGACGAACTTGGCCTGT	59.4/57.3	43
	AflS4‐BIP	TGTTGACCATTATGTGCGCC‐TTTTT‐CATGGCGACGAACTTTGG	57.3/56.0	43
	AflS4‐LF	AACGCGCCTGATGCTGC	57.6	17
	AflS4‐LB	TCGAGGGGCATGGTGGA	57.6	17

In silico analysis showed that the primer set AFLS4 aligned with high identity to the *nor1* gene sequences of *A. flavus* and *A. parasiticus* . However, partial sequence similarity was also observed with *A. nomius* and *A. oryzae* , indicating potential cross‐reactivity. Therefore, further in vitro validation is necessary to confirm the assay's specificity and exclusivity for the target species.

### 3.3 Specificity and Sensitivity

**FIGURE 3:**
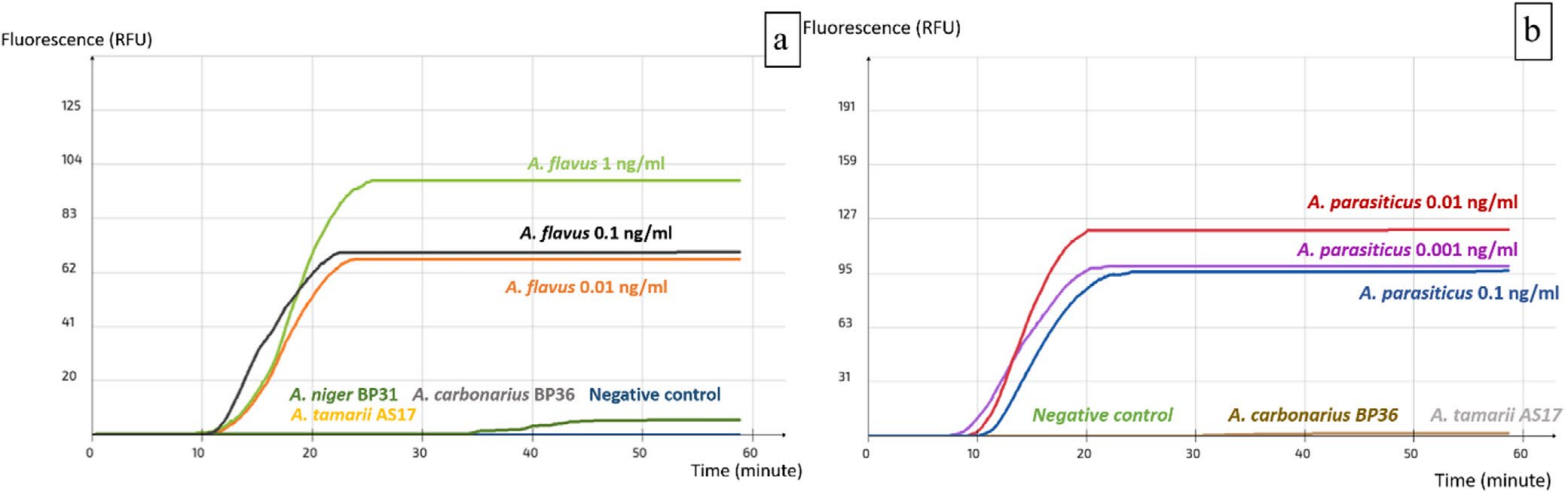
Specificity and sensitivity of the LAMP assay AFLS4 (a) *A. flavus* and nontarget species (b) *A. parasiticus* and nontarget species.

To determine the accuracy of results obtained with the set AFLS4 as well as its sensitivity level in terms of pg of purified DNA, the primary tests were performed on pure DNA of aflatoxin producers including *A. flavus* BP50 and *A. parasiticus* AP1. A nonproducing strain of *A. tamarii* AS17 as well as the two strains of *A. carbonarius* BP36 and *A. niger* BP31 (both OTA producers) were also considered as nontarget species (Table 4). The reaction's optimal temperature was 63°C. This set's detection level was 0.01 ng/uL, which is equivalent to 10 pg/mL and 0.03 pg/reaction (Figure 3). Furthermore, the test demonstrated a specific detection of *A. flavus* and *A. parasiticus* among other species (Figure 3).

**TABLE 4:** Results of LAMP test on target and nontarget species.

Target species	Strain	LAMP result
*A. flavus* (120 strains)	PGA2‐53 PGA57‐69 PGA71 PGA73‐91 PGA 93–117 BP50 BP51 BP53 AS3 AS6‐10 AS15	Positive
*A. parasiticus* (5 strains)	PGA54 PGA56 PGA70 PGA72 PGA 92	Positive
**Nontarget species**	**Strain**	**LAMP result**
*A. niger* (7 strains)	BP4 BP8 BP10 BP16 BP31 BP34 BP35	Negative
*A. tubingensis* (3 strains)	AS17 BP29 BP33	Negative
*A. tamarii* (4 strains)	PGA1 PGA31 PGA88 AS17	Negative
*Penicillium digitatum* (1 strain)	A1P1	Negative
*A. carbonarius* (3 strains)	BP36 A. C1 A.C2	Negative

### 3.4 Primer Dehydration and Stability

**FIGURE 4:**
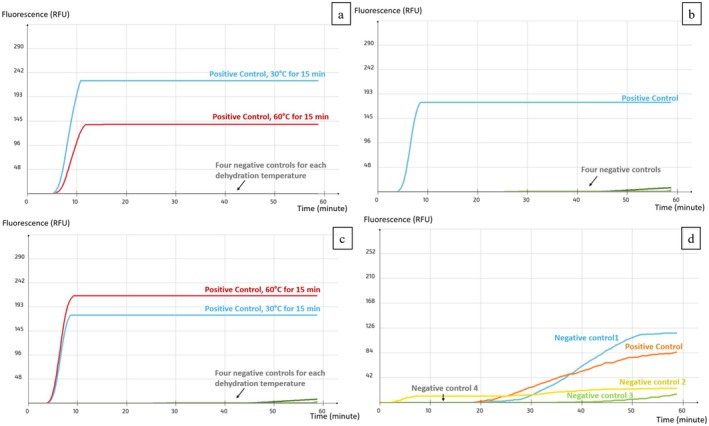
Stability test (a) Freshly dehydrated primers, (b) Freshly prepared primer solutions, (c) Dehydrated primers stored for 15 days at RT, and (d) Primer solutions stored for 15 days at RT.

Primer dehydration enabled the preservation of primer stability, thereby extending their shelf life. Primers tested immediately after dehydration produced results comparable to those obtained from freshly prepared solutions at both temperatures (Figure 4). After 15 days, dehydrated primers continued to yield consistent results, whereas stored solutions failed to provide accurate outcomes. A dehydration temperature of 30°C was selected for future experiments, as it offers both ecological and economic advantages.

**FIGURE 5:**
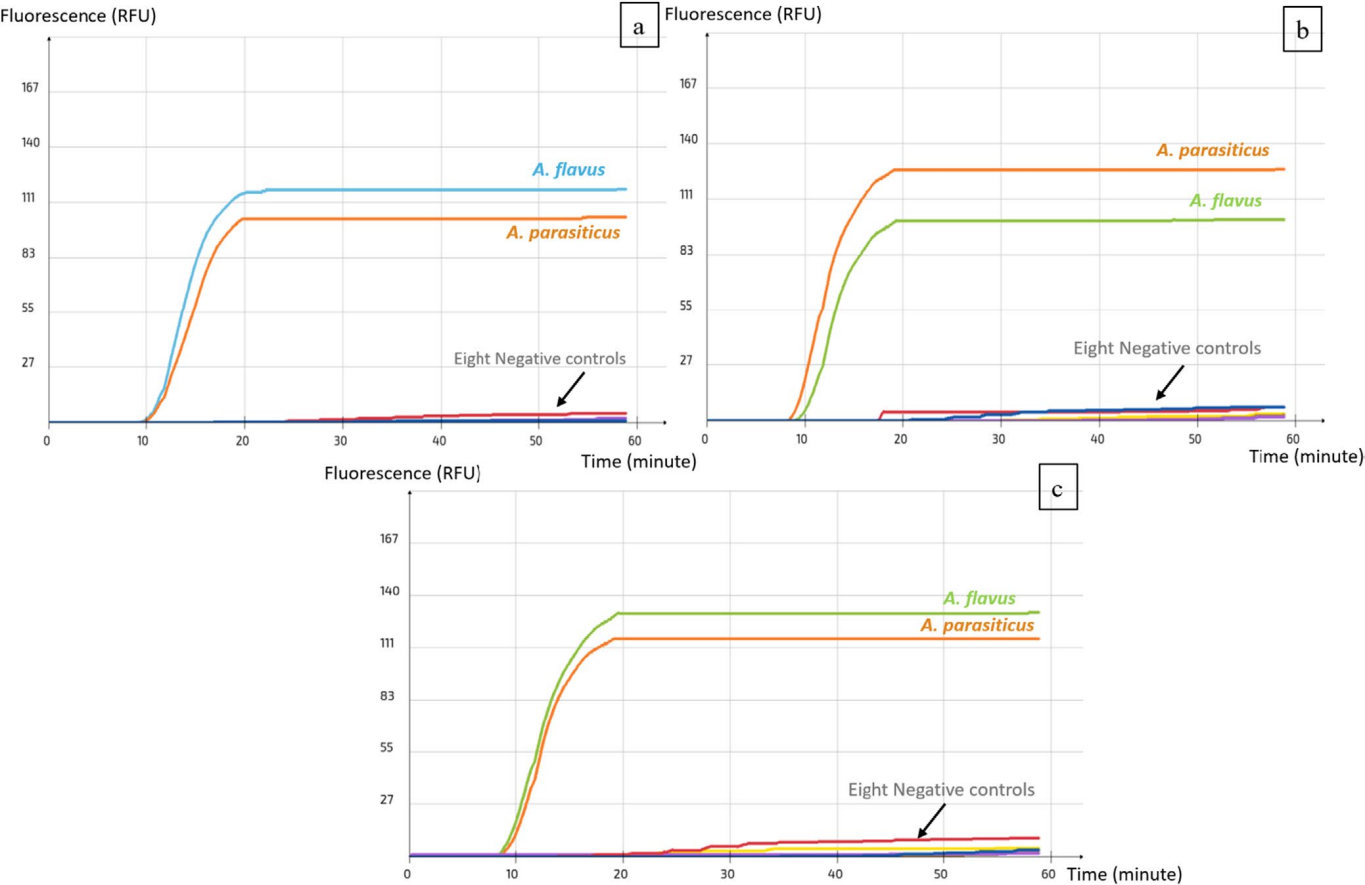
Stability of dehydrated primers after storage for 45 days at (a) 4°C, (b) RT (25°C ± 2°C), and (c) 35°C.

The stability of primers dehydrated at 30°C was evaluated over a 45‐day period under three different storage conditions: 4°C, RT (25°C ± 2°C), and 35°C, with assessments conducted at 15‐day intervals. The dehydrated primers remained stable and consistently produced accurate results across all storage temperatures throughout the testing period, with the final assessment at 45 days correctly detecting only the positive samples (Figure 5). These findings highlight the robustness of the dehydration process and confirm the primers' suitability for use in environments lacking controlled storage conditions, including during transportation or in resource‐limited settings.

### 3.5 Evaluation of the LAMP Assay on Food Material

**FIGURE 6:**
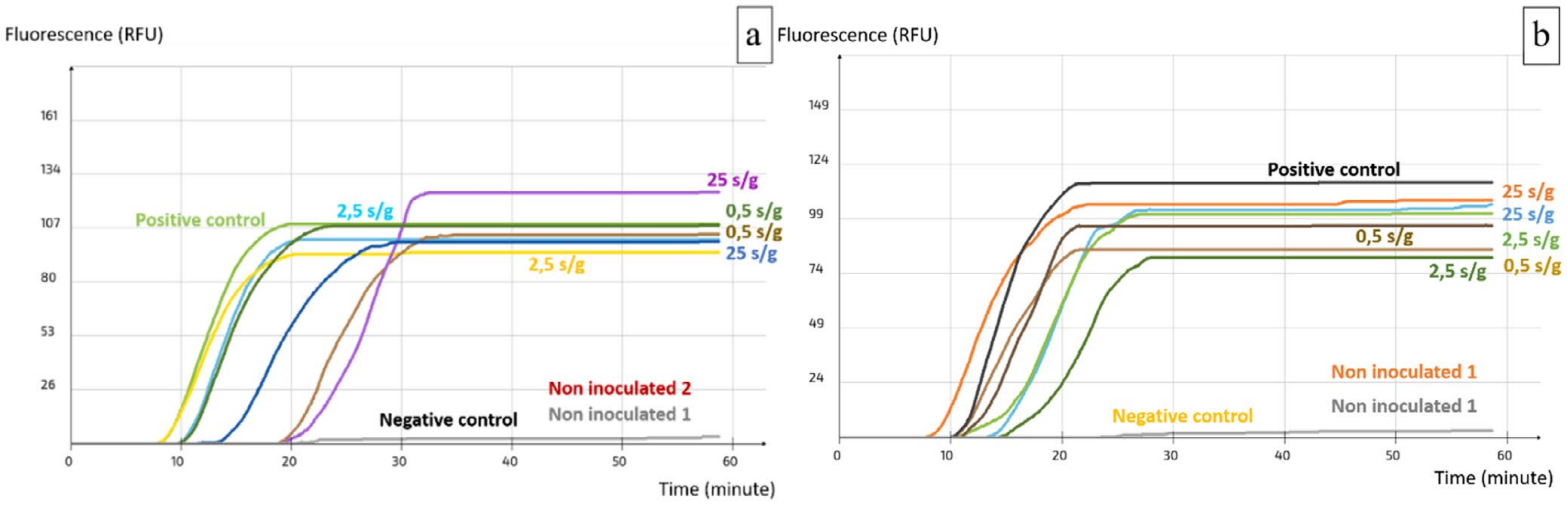
Implementation of the LAMP assay AFLS4 on pistachio nuts inoculated with spore suspensions of different concentrations. (a) *A. flavus* and (b) *A. parasiticus* .

Given that LAMP's advantage is its ability to detect crude DNA directly from food samples, our set was tested on artificially inoculated pistachio samples after they underwent a one‐step crude DNA extraction. The pre‐enrichment phase allowed spore germination and mycelium growth, thereby ensuring a sufficient quantity of DNA to be detected by the primers. LAMP set's lowest tested detection level was determined at 0.5 spore/g for both *A. flavus* and *A. parasiticus* (Figure 6a,b). Although the development of a successful amplification is often threatened by food inhibitors' disturbance of the reaction, in this case, Whatman filter paper was used to eliminate most inhibitors. Furthermore, the LAMP set did not detect any of the non‐inoculated samples considered as a control (Figure 6).

**FIGURE 7:**
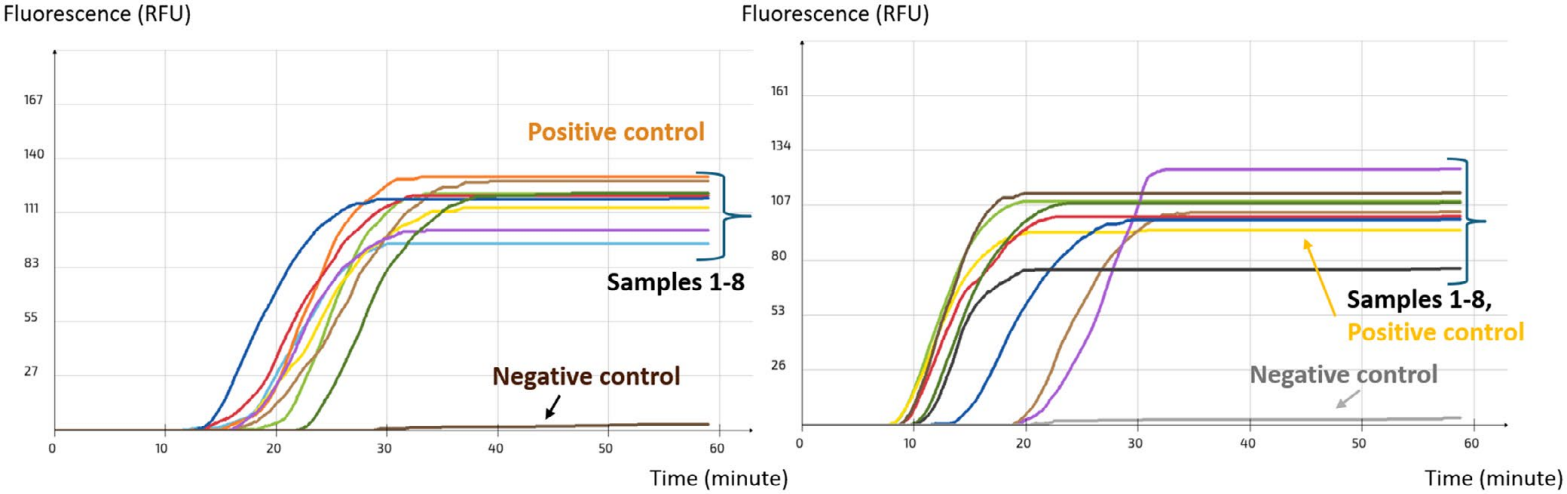
Application of the LAMP assay on samples of pistachios from diverse varieties and sources inoculated with (a) *A. flavus* and (b) *A. parasiticus* .

The applicability of the assay was successfully demonstrated using the air‐dried primer set on inoculated samples of various pistachio varieties sourced from the field, storage facilities, and local markets. Amplification signals were observed in all inoculated samples for both fungal species (Figure 7), confirming the set's effectiveness in detecting low concentrations of fungal spores directly from food matrices, after a one‐step crude DNA extraction. No amplification was observed in the negative control tubes containing nuclease‐free water, confirming the absence of false positives (Figure 7).

## 4 Discussion

Pistachio nut production and commercialization are often threatened by aflatoxins, which are harmful mycotoxins produced by *Aspergillus* species mainly from the *Flavi* section (Campbell et al. 2003; Perrone and Gallo 2017). Due to aflatoxins' toxicity, most countries strictly regulate their levels. Detecting aflatoxigenic species in nuts is therefore important, as their presence indicates a significant risk of aflatoxin contamination.

Aflatoxins are mainly produced by *A. flavus* and *A. parasiticus;* moreover, *A. nomius* has been reported to contribute to aflatoxin contamination in various nuts, including pistachios (Doster et al. 2009) and Brazil nuts (Olsen et al. 2008). This study further confirmed the high occurrence of *A. flavus* and *A. parasiticus* in pistachios sampled in Italy as well as their significant capacity to produce AFB1 through HPTLC and HPLC analyses. It has been reported that approximately 30%–40% of *Aspergillus flavus* strains are incapable of producing aflatoxins (Alaniz Zanon et al. 2018; Horn and Dorner 1999). However, in the case of pistachios, this proportion appears to be lower, with previous studies suggesting that the majority of *A. flavus* strains isolated from pistachios are capable of mycotoxin production (Fernane et al. 2010; Moghadam et al. 2020). In our study, 100% of the *A. flavus* isolates produced aflatoxins, which can be attributed in part to the use of optimized culture conditions specifically designed to stimulate aflatoxin biosynthesis. It is important to note that this result may not directly reflect aflatoxin production on pistachios under natural conditions. Future studies assessing toxin production directly on pistachio substrates would be valuable for understanding the true risk in real‐world scenarios.

*A. flavus* and *A. parasiticus* were the two species taken into consideration when designing a LAMP primer set able to detect aflatoxigenic aspergilli associated with pistachios. Available sequences in the NCBI database of these species were included in the primer design and alignment steps. *A. flavus* accounts for the majority of aflatoxigenic fungi found on pistachios, as demonstrated by previous studies (Hua et al. 2012; Marín et al. 2012; Mellikeche, Casini, et al. 2024) and here confirmed (section 3.1). However, in the Mediterranean area, certain factors, such as climate change, have led to an increasing presence of *Aspergillus* spp. sec. *Flavi* other than *A. flavus* capable of producing aflatoxins, such as *A. parasiticus* . Therefore, species identification alone is less important than detecting the actual ability of a fungus to produce aflatoxins. To address this, we designed a LAMP primer set that directly detects both of these aflatoxigenic aspergilli regardless of the specific species, ensuring a rapid, targeted approach. By focusing on aflatoxin production rather than species identification, this method provides a more efficient and rapid tool for detecting contamination risks.

The primers were evaluated in silico and demonstrated high compatibility with the target species. This was further confirmed in vitro, where a real‐time LAMP device detected fluorescence signals when testing their DNA. The in silico analysis also indicated potential cross‐reactivity with *A. nomius* and *A. oryzae* . Detection of *A. nomius*, an aflatoxin producer, would be advantageous for the assay's intended purpose. In contrast, *A. oryzae* is primarily used in food fermentation and, to the best of our knowledge, has not been isolated from pistachios. Nevertheless, additional testing of the LAMP assay on these species is necessary to better evaluate its specificity.

Primer dehydration was a crucial step in this protocol, aimed at simplifying its application by eliminating the need to prepare primer and dye mixtures for each use, while also extending primer shelf life. Dehydrated primers produced results comparable to freshly prepared solutions when tested immediately, and maintained this performance after 15 days of storage, unlike liquid‐stored solutions, which degraded over time. To assess long‐term stability, primers dehydrated at 30°C were stored under three different conditions: 4°C, RT (25°C ± 2°C), and 35°C, and tested at 15‐day intervals over a 45‐day period. In all conditions, the dehydrated primers consistently generated accurate results, detecting only positive samples by day 45. These findings highlight the value of primer dehydration in preserving the set during transport, without requiring cold storage, and in facilitating protocol application in resource‐limited settings.

Niessen et al. (2018) developed a *nor1*‐targeted LAMP assay for group‐specific detection of aflatoxin‐producing *Aspergillus* section *Flavi*. In that work, primers were used in liquid form without stabilization or dehydration, which may limit long‐term applicability. Results were read by colorimetric indicators (neutral red), fluorescence (calcein/UV), real‐time fluorimetry, and confirmed by agarose gel electrophoresis. In our study, the result reading method was simplified by performing it with an easy‐to‐use portable real‐time device that can be attached to a phone or tablet, and primer dehydration was incorporated to improve stability and extend shelf life. Under our conditions, the limit of detection was 0.03 pg of genomic DNA, compared with 9.03 pg reported by Niessen et al. (2018). Other LAMP assays targeting aspergilli were applied to nuts such as Brazil nuts (Luo et al. 2012) peanuts (Al‐Sheikh 2015) and hazelnuts (Ortega et al. 2020). To the best of our knowledge, no previous research tested them on pistachios despite their susceptibility to such contaminations. This could be due to their physiochemical properties, such as their high lipid content and their color that might interfere with the fluorescence signal. In this study, the designed primers were incorporated into a protocol for direct detection of aflatoxin‐producing species from pistachios. Overnight pre‐enrichment in PDB allowed the spores to germinate and produce mycelium, which resulted in more DNA material for detection. Furthermore, filtration allowed for eliminating debris and reaction inhibitors. This approach enabled the LAMP assay to achieve very high sensitivity, with a capability of detecting contamination levels as low as 0.5 spores/g. Notably, comparable results can be achieved by increasing the initial spore quantity, eliminating the need for the pre‐enrichment step. Furthermore, the protocol successfully detected *A. flavus* and *A. parasiticus* across diverse pistachio samples, which confirms the assay's reliability and broad applicability.

## 5 Conclusion

The research community's interest in LAMP is increasing due to the technique's efficiency and simplicity, which facilitates its use in mass screenings to detect food contaminants. This study further confirmed LAMP's efficacy and accuracy at detecting some of the most toxic and widespread food contaminants: aflatoxigenic aspergilli. In the future, similar assays are expected to be developed for other susceptible nuts, along with their incorporation in kits for onsite analysis. These kits will enable storage workers to perform mass testing independently, without the need for expert intervention, to ensure more efficient and accessible food safety monitoring.

## Author Contributions

**Wanissa Mellikeche:** conceptualization (lead), methodology (lead), writing – original draft (lead). **Alessandra Ricelli:** conceptualization (supporting), supervision (lead), writing – review and editing (supporting). **Marah Abukhmaish:** methodology (supporting), writing – review and editing (supporting). **Rocco Caracciolo:** methodology (supporting), supervision (supporting). **Marilita Gallo:** supervision (supporting). **Carlotta Casini:** writing – review and editing (supporting). **Giancarlo Colelli:** funding acquisition (lead), supervision (lead). **Anna Maria D'onghia:** funding acquisition (lead), supervision (supporting), writing – review and editing (supporting). **Franco Valentini:** funding acquisition (supporting), supervision (supporting).

## Conflicts of Interest

Rocco Caracciolo was employed by Enbiotech SRL. The remaining authors declare that the research was conducted in the absence of any commercial or financial relationships that could be construed as a potential conflicts of interest.

## Data Availability

The raw data supporting the conclusions of this article will be made available by the authors on request.
